# Engineered mesenchymal stromal cells with interleukin-1beta sticky-trap attenuate osteoarthritis in knee joints

**DOI:** 10.3389/fcell.2025.1559155

**Published:** 2025-04-08

**Authors:** Christopher Kim, Biao Li, Sayaka Nakamura, Eric J. Neely, Jason S. Rockel, Tatiana Oussenko, Puzheng Zhang, Mohit Kapoor, Andras Nagy

**Affiliations:** ^1^ Lunenfeld Tanenbaum Research Institute, Sinai Health System, Toronto, ON, Canada; ^2^ Schroeder Arthritis Institute, Krembil Research Institute, University Health Network, Toronto, ON, Canada; ^3^ Department of Obstetrics and Gynaecology and Institute of Medical Science, University of Toronto, Toronto, ON, Canada; ^4^ Australian Regenerative Medicine Institute, Monash University, Melbourne, VIC, Australia

**Keywords:** osteoarthritis, sticky biologics, cell-based gene therapy, interleukin-1beta, interleukin-1 receptor 2, mesenchymal stromal cell

## Abstract

Osteoarthritis (OA) is a common chronic inflammatory joint disease, in which innate immunity plays a pivotal role in pathogenesis. Anti-interleukin-1(IL-1) therapies have shown inconsistent results in clinical trials, potentially due to a mismatch in the spatial and temporal dynamics of interleukin-1beta (IL-1β) production and therapeutic interventions. To address this issue, we developed a novel IL-1β “sticky-trap” utilizing cell and gene-based technologies from our lab and evaluated its efficacy in reducing osteoarthritis progression using a murine destabilization of the medial meniscus (DMM) OA model and a compact bone-derived mesenchymal stromal cell (MSC)-based gene expression system. The extracellular domain of interleukin-1 receptor 2 (IL1R2) was employed to design the sticky IL1R2 trap (stkIL1R2). A murine compact bone-derived MSC line was engineered for gene delivery. Although stkIL1R2 was undetectable in the engineered MSC supernatants by enzyme-linked immunosorbent assay (ELISA) and Western blot, it was localized on the cell surface and extracellular matrix (ECM) and demonstrated specific binding to IL-1β using a fluorescent protein-fused binding assay. Doxycycline (Dox)-induced expression of stkIL1R2 significantly inhibited lipocalin-2 (LCN2) expression which is a biomarker of IL-1β activity. For *in vivo* experiments, 5 × 10^4^ Dox-inducible stkIL1R2f expressing MSCs were injected into the knee joints of DMM mice. Bioluminescence imaging revealed MSC survival in the knee joints for up to 7 weeks post-injection. Histological analyses at 10 weeks post-injection, including Safranin-O and Masson trichrome staining, showed that stkIL1R2 treated joints exhibited significantly less cartilage degradation and synovitis compared to controls, as assessed by Osteoarthritis Research Society International (OARSI) scoring of the femur, tibia, and synovium. Moreover, stkIL1R2 treatment reduced matrix metalloproteinases-13 (MMP-13) positive cells and collagen type II degradation in the affected joints. In conclusion, we developed a MSC line expressing an inducible IL1 sticky-trap, which localized to the cell surface and ECM and specifically bound IL-1β. These engineered MSCs survived in normal and DMM knee joints for up to 7 weeks and significantly delayed OA progression and inflammation in the murine model. This study introduces a promising therapeutic approach to combat OA progression.

## 1 Introduction

Osteoarthritis (OA) is a chronic, degenerative, whole-joint disease characterized by the breakdown of articular cartilage, osteophyte formation, remodeling of subchondral bone, and inflammation of the synovial membrane (Pelletier et al., 2001). An imbalance of chondrocyte homeostasis leads to the progressive degradation of cartilage extracellular matrix, involving pro-inflammatory cytokines and matrix metalloproteinases (MMPs) (Pelletier et al., 2001). OA stands as a leading cause of disability, pain, loss of function and decreased quality of life (QOL) in the elderly (Hunter et al., 2014).

Increasing evidence suggests that OA is a low-grade inflammatory condition of the joint (Robinson et al., 2016; Scanzello, 2017a). Notably, the interaction between cartilage and the synovial membrane is critical in sustaining this chronic low-grade inflammation (Chou et al., 2020; Hamasaki et al., 2021; Sanchez-Lopez et al., 2022; Scanzello and Goldring, 2012). The term “low-grade inflammation” distinguishes OA from inflammatory arthritis like rheumatoid arthritis (RA). Unlike RA, where inflammation primarily arises from adaptive immunity, OA’s inflammation stems from innate immunity, either systemic or local (Knights et al., 2023; Roover et al., 2023). The pro-inflammatory cytokine interleukin-1beta (IL-1β) plays a key role in innate immunity (Hoseini et al., 2018; Im and Ammit, 2014; Keyel, 2014) and has been implicated in both preclinical and clinical OA conditions (Krenytska et al., 2023; Panina et al., 2017; Wang et al., 2019). However, anti-interleukin-1 (IL-1) therapies adapted from RA treatments have shown limited success in OA (Chevalier et al., 2009; Cohen et al., 2011; Fleischmann et al., 2019; Kloppenburg et al., 2018; 2019), with debates surrounding IL-1β′s role in OA (Chevalier et al., 2009; Cohen et al., 2011; Fleischmann et al., 2019; Kloppenburg et al., 2018; 2019). These debates were clarified by the Canakinumab Antiinflammatory Thrombosis Outcome Study (CANTOS), where IL-1β inhibition significantly reduced the need for hip or knee replacements (Ridker et al., 2017; Schieker et al., 2020). The variability in preclinical and clinical outcomes can be attributed to OA’s inflammatory heterogeneity, its diverse phenotypes and endotypes (Bosch et al., 2024; Chevalier et al., 2013), and the short half-life of IL-1β in serum (Kudo et al., 1990; REIMERS et al., 1991). This underscores the need for precise timing and delivery strategies for anti-IL-1 therapies.

Presently, OA remains incurable and no approved medications, biological therapy, or procedure cures or prevents the progressive destruction of the joint. Present treatment options primarily offer symptomatic relief rather than preventative or regenerative results. There is an urgent call to innovate, validate, and explore new biological therapeutics. Among these, cell-based therapies involving the injection of MSCs to the osteoarthritic knee joint have surfaced as a promising solution to address this clinical challenge.

Our lab has pioneered a novel approach to express local-acting biologics, termed “sticky-trap” technology, which was previously successful in trapping vascular endothelial growth factor (VEGF) in the eye (Michael et al., 2014). Building on this success, we developed a IL-1β sticky-trap using the extracellular domain of IL-1 receptor 2 (IL1R2) and employed isogenic mouse compact bone-derived MSCs as carriers. This study explores whether this cell-based therapy can mitigate inflammation and slow OA progression in a murine DMM model. Our hypothesis is that intraarticular injection of local acting IL-1β sticky trap expressing MSCs could reduce inflammation and attenuate the progression of OA.

## 2 Methods

### 2.1 Mouse studies

Mouse studies were conducted following a protocol approved by the Institutional Animal Care and Use Committee of The Toronto Phenogenomics (TCP, Toronto)(AUP #25–0308H). Mice were housed in a room with controlled temperature (21°C–23°C), humidity (40%–60%), and a light cycle consisting of 12 h of light (starting at 07:00) and 12 h of dark (starting at 19:00). Mice were kept in individual ventilated cages (Techniplast Greenline GM500 cage, LABEX Inc., MA, United States) and provided with quality water via an automated watering system (Avidity Science, WI, United States) with a sterile quick-disconnect Avidity Science water valve. They were fed with extruded pellets from Envigo (Inotiv Inc., WI, United States). C57BL/6J mice were purchased from TCP and maintained in the mouse room.

### 2.2 MSC isolation and culture

MSCs were isolated from compact bone as per Zhu et al. (2010). Briefly, a 3-week-old C57BL/6J mouse was euthanized by cervical dislocation. The carcass was rinsed in 70% ethanol for 3 min, and femurs were dissected in a sterile 100-mm dish. Bone marrow was flushed out using 5 mL of α-MEM (Gibco, Thermo Fisher, cat. no. 12571063), supplemented with 0.1% penicillin/streptomycin (P/S) and 2% FBS (Multicell, Wisent Inc., cat. no. 080150) until the bones became clear. The femurs were then chopped into 1–3 mm^3^ pieces using scissors. Bone chips were digested in 3 mL of α-MEM containing 10% FBS and 1 mg/mL collagenase II (Sigma, cat. no. C6885) and incubated at 37°C with shaking (200 rpm) for 2 h. The bone chips were washed three times with 5 mL of α-MEM and seeded into a 100-mm sterile dish with 10 mL of DMEM/F12 medium (Gibco, Thermo Fisher, cat. no. 11330-032) supplemented with 10% FBS, 1% P/S, and 10 ng/mL bFGF (PeproTech, cat. no. 100-18B). Cells were incubated at 37°C in a 5% CO_2_ incubator for 3 days without disturbance. The medium was changed every 3 days until confluence. These cells were defined as passage 0 (P0). MSCs were cultured in DMEM/F12 with 10% FBS, 10 ng/mL bFGF, and 1% P/S. Cells were passaged with 0.25% trypsin-EDTA and cryopreserved at −80°C for 1 day before being transferred to liquid nitrogen.

### 2.3 FACS sorting

Cells were harvested by trypsin digestion, washed twice with cold PBS, and incubated with antibodies on ice for 10 min. After washing, cells were filtered and analyzed by flow cytometry using a Beckman Coulter Gallios and BD Fortessa X-20 flow cytometer. Data were analyzed using Beckman Coulter Kaluza and FlowJo software (BD Biosciences, ver. 10.10.0). Antibodies used for flow analysis were listed in Supplementary Table S1.

### 2.4 Quantitative PCR

Freshly harvested cells were used for total RNA purification with Trizol regent (cat. no. 15596-026, Invitrogen). The purified RNA was dissolved in DEPC-treated water (Diethyl pyrocarbonate) (cat. no. D5758-25 mL, sigma). RNA concentration was quantified by Nanodrop 1000 spectrophotometer (Thermo Scientific). A total of 1 μ g of RNA was reverse transcribed to cDNA using the iScript cDNA Synthesis Kit (cat. no. 1708891, Bio-Rad) following the manufacturer’s instructions. For amplification, 1 μ l of 1:100 diluted cDNA sample was applied in the SsoAdvanced Universal SYBR green supermix (cat. no. 1725271, Bio-Rad) in a total reaction volume of 5 μ l, prepared in a 384-well plate (cat. no. HSP3805, Bio-Rad). The reactions were were run on a DNA amplifier (CFX384 Real-Time system, C1000 Thermal cycler, Bio-Rad). All samples were analyzed in duplicate. Relative quantification of gene expression was calculated using the comparative Ct method (Schmittgen and Livak, 2008). The ΔCt value was determined by subtracting the Ct value of the house keeping gene from respective respective target gene Ct value for each sample. Relative expression levels were calculated as 2^−ΔCt^, and incremental changes in target gene expression were determined as 2^−ΔΔCt^. Primers used in this study are listed in the Supplementary Table S2.

### 2.5 Generation of enhanced luciferase expression cell line

The PB-CAG-e-Luciferase-IRES-eGFP plasmid was generated in our lab and used to transfect mouse MSCs. MSCs (2 × 10^6^) were plated in a 100-mm plate and incubated overnight at 37°C in 5% CO_2_. When cells reached 70%–90% confluence, they were transfected using jetPRIME transfection reagent (Poly-Plus Transfection, France, cat. no. #114-15) according to the manufacturer’s instructions. Briefly, 10 μg of DNA was diluted in 500 μL jetPRIME buffer, mixed with 20 μL jetPRIME reagent, and incubated at room temperature for 10 min. The mixture was then added to the cells. The plate was incubated at 37°C in 5% CO_2_ for 24–48 h. Gene expression was confirmed by the GFP signal observed under fluorescence microscopy.

### 2.6 Cell differentiation

MSC differentiation reagents were purchased from LONZA (www.lonza.com). Adipocyte, chondrocyte, and osteocyte differentiation protocols were followed as recommended by the manufacturer. For each differentiation protocol, the same number of cells were cultured in standard MSC medium for the same duration as the control group.

### 2.7 Bioluminescence imaging (BLI)

Bioluminescent signals from e-luciferase and luciferin reactions in cells were acquired using the IVIS Lumina III BLI System (PerkinElmer, Guelph, Canada; Part No. CLS136334). Mice were injected with 200 μg of luciferin (Promega, Madison, United States, cat. no. P1043) in 100 μL PBS via intraperitoneal injection, anesthetized with isoflurane USP (Piramal Critical Care Inc., Bethlehem, United States, cat. no. DVM-102190), and imaged. Care was taken to remove hair from the knees of the mice before imaging.

### 2.8 Generation of inducible gene expression plasmids

The extracellular domain cDNA sequence, including the signal peptide of murine IL1R2 (nt1-1065), was used for constructing the short trap IL1R2 (sIL1R2) and sticky trap IL1R2 (stkIL1R2) plasmids. Additional domains included a GS linker, IgG1-CH3 domain, heparin-binding domain (exons 6 and 8), a Flag tag, and an His tag (x8). Inducible expression plasmids for stkIL1R2 and sIL1R2 were generated using Invitrogen Gateway Technology. The target gene cDNA sequences were flanked by att-B and att-P sequences (Eurofins Genomics, Toronto, Canada). Synthesized DNAs were subcloned into a pDNOR221 plasmid via BP reaction, then transformed into One Shot TOP10 chemically competent *E. coli* (Invitrogen, cat. no. 4040-10). After enzyme digestion, the targeted plasmid was confirmed by DNA sequencing (ACGT Corp., Toronto, Canada). The confirmed plasmid was then used for an LR reaction with the final targeting plasmid (PB-teto-GW-IRES-GFP/mCherry). The plasmids were transformed into *E. coli*, and the final plasmids were confirmed by enzyme digestion. Colonies were maxi-prepped (Qiagen, Hilden, Germany) for high-quality DNA. This protocol was also used for other expression plasmids in this study. IgG and stk plasmid controls in protein generation studies using HEK293 cells were kindly donated by coauthor, Dr. Eric Neely.

### 2.9 Western blot analysis

Tissues and cells were lysed in RIPA buffer (50 mM Tris-HCl, pH 7.4, 150 mM NaCl, 1% Triton X-100, 1% sodium deoxycholate, 0.1% SDS, 1 mM EDTA) with protease inhibitor mix (Sigma). Homogenization was performed using a TissueLyser (Bio-Rad), followed by centrifugation at 13,200 rpm for 15 min at 4°C. The supernatant was collected for protein analysis. Protein concentration was measured by the Pierce BCA protein assay kit (Thermo Scientific, cat. no. 23225).

20 μg of protein was resolved on a NuPAGE 4%–12% Bis-Tris SDS-PAGE gel (Thermo Scientific, cat. no. NP0336) and transferred to a PVDF membrane using an iBlot 2 PVDF mini stack transfer kit (Thermo Scientific, cat. no. IB24002). After blocking with 5% non-fat milk, the membrane was incubated with primary antibody overnight at 4°C, followed by incubation with HRP-conjugated secondary antibody. Proteins were detected using the Amersham ECL prime Western blotting detection reagent (GE Healthcare Life Sciences, cat. no. RPN2232). Images were acquired and processed using a ChemiDoc XRS + system (Bio-Rad).

Antibodies used: rabbit monoclonal to lipocalin-2 (LCN2) (Abcam, cat. no. 216462), goat anti-rabbit IgG H&L HRP secondary antibody (Abcam, cat. no. ab7090), and mouse monoclonal anti-β-Actin antibody (Sigma, cat. no. A3854).

### 2.10 ELISA

To assess IL1R2 expression, 5 × 10^5^ inducible sIL1R2 and stkIL1R2 expressing MSCs were plated into a 6-well plate, with Dox either present or absent. The cells were cultured at 37°C in 5% CO_2_ for 72 h, and the supernatants were collected for ELISA analysis. Mouse IL1R2 was quantified using the mouse IL1R2 ELISA kit (cat. no. RD-IL1R2-mu, RedDot Biotech Inc., Kelowna, Canada) following the manufacturer’s protocol. Briefly, samples were diluted according to the kit’s instructions using calibrator diluent and incubated for 2 h at room temperature (RT) with the primary antibody-coated 96-well strips. After four washes with washing buffer, the samples were incubated with the HRP-conjugated secondary antibody for 2 h at RT. Following an additional four washes, substrate solution was added for 30 min, and the reaction was stopped with the provided stop solution. Optical density (OD) was measured at 450 nm and 540 nm using an EnSpire 2300 multilabel reader (PerkinElmer, ON, CA). A standard curve was constructed using the provided mouse LCN2 standard, and OD values were corrected by subtracting OD540 from OD450. The corrected OD values were then used to calculate IL1R2 concentration based on the standard curve.

### 2.11 Protein generation

HEK-293F cells were used for protein production. The cells were cultured in Freestyle 293 medium (cat. no. 12338-018, Gibco) supplemented with 2.5 mM sodium butyrate (cat. no. 303410, Sigma) at 37°C and 5% CO_2_, shaking at 1.5 mm/s for 3 days. pH and glucose levels were monitored daily to maintain a pH of 6.8 and glucose concentration between 18 and 27 mmol/L. After 3 days, the cells were spun down, and the supernatant was collected and stored at 4°C. The cells were then resuspended in fresh medium without sodium butyrate and cultured for another 3 days. The supernatants from both collections were combined for protein purification. To remove cellular debris, the supernatants were filtered through a 0.45 µm Nalgen filter (cat. no. 166-0045, Thermo Scientific), then incubated with Heparin Sepharose 6 Fast Flow beads (cat. no. 17-0998-01, Qiagen) at 4°C overnight. The beads were packed into a column the following day, eluted with NaCl, and the eluates were bound to a Ni-NTA column (cat. no. 30410, Qiagen) and eluted with imidazole (cat. no. 15513–25 g, Sigma). The eluates were concentrated to 2–3 mL using an Amicon Ultra-15 centrifugal filter (EMD Millipore, United States) and desalted with a PD10 column (cat. no. 17-0851-01, GE Healthcare Life Sciences). Finally, proteins were eluted from the PD10 column with 3 mL storage buffer (5% D-Mannitol, 5% D-Trehalose dihydrate in PBS) (Sigma). Protein concentration was determined using Bradford reagent (cat. no. 15513, Bio-Rad).

### 2.12 Protein binding assay

To generate the IL-1β sticky-trap, we constructed two plasmids: PB-tetO-stkIL1R2-mCherry-IRES-puromycin (which expresses stkIL1R2-mCherry under Dox induction) and PB-tetO-IL-1β-GFP-IRES-puromycin (which expresses IL-1β-GFP under Dox induction) (Figure 3A). The stkIL1R2-mCherry plasmid was transfected into R61MSC and HEK-293F cells, and protein expression was induced by Dox. Fluorescent signals were monitored using a fluorescent microscope. The IL-1β-GFP plasmid was transfected into HEK-293F cells, and the supernatant was collected for IL-1β-GFP protein purification.

For the sticky-trap assay, 7.5 × 10^4^ cells expressing IL-1β sticky-trap were plated onto coverslips in a 6-well plate and incubated with Dox (1.5 μg/mL) for 48 h. The medium was removed, and the cells were washed three times with PBS, then fixed with 4% paraformaldehyde (PFA) (cat. no. J61899, Alfa Aesar) in PBS (unpermeabilized) for 10 min at RT. After fixation, cells were washed three times with PBS and blocked for 1 h at RT using blocking buffer (2% BSA in PBS). The cells were washed three times with PBS and incubated with IL1β-GFP protein (5 ng/mL) for 1 h at RT. After washing, coverslips were mounted with VECTASHIELD Antifade Mounting Medium with DAPI (cat. no. H-1200-10, Vector Laboratories) and imaged using a Carl Zeiss LSM 780 confocal microscope.

### 2.13 DMM mouse knee OA model

The DMM model was used to induce OA in male C57BL/6J mice (10 weeks old). The procedure followed Glasson et al. (2007). Mice were anesthetized with isoflurane and the right hind leg was disinfected with 70% ethanol. A 3 mm longitudinal incision was made over the distal patella to the proximal tibial plateau. The joint capsule medial to the patella tendon was opened with micro-iris scissors, and the medial meniscotibial ligament (MMTL) and medial meniscus (MM) were visualized and transected with micro-iris scissors. Transection was confirmed by lifting the medial meniscus from the tibial plateau. The joint capsule and skin were sutured closed. Sham surgeries were performed with identical aseptic technique, but without ligament transection. DMM and sham surgeries were performed by the same surgeon. Depending on the experimental group, mice were administered Dox chow (Envigo, green) post-surgery.

### 2.14 Histology

Following euthanasia, freshly dissected mouse hind limbs without skin were fixed overnight in 4% paraformaldehyde/PBS (cat. no. J61899, Alfa Aesar). Tissues were then decalcified in 10% EDTA for 1 week, embedded in paraffin, sectioned sagittally, and stained with H&E, Safranin-O, Fast Green, and Masson’s Trichrome. For Immunohistochemistry (IHC), paraffin slides were deparaffinized in xylene, followed by alcohol treatment. Antigen retrieval was performed using 10 mM citrate buffer (pH 6.0). Slides were blocked with Dako protein block for 10 min at RT, then incubated overnight at 4°C with primary antibodies. After washing with PBS-T, slides were incubated with HRP-conjugated secondary antibody for 1 h at RT. Following washes, slides were developed using 3,3′-diaminobenzidine (DAB), and counterstained with H&E. OARSI score was used to quantify the pathological changes in femur, tibia and synovial membrane. OARSI scoring was performed by two independent researchers using the OARSI semi-quantitative scoring system (Glasson et al., 2010). Both scorers were blinded to the experimental groups.

### 2.15 Statistical analysis

Data were presented as mean ± SEM. Data were first analyzed using an F-test to determine if the variances were significantly different or not. Then, a two-tailed unpaired Student’s t-test, accounting for equal or unequal variance, was performed to compare two independent groups, p < 0.05 was recognized as a significant difference, with all analyses performed in Microsoft Excel and Prism 10 (GraphPad Software Inc). Figures were generated using Prism 10.

## 3 Results

### 3.1 Isolation and primary culture of compact bone derived MSC cell line

**FIGURE 1:**
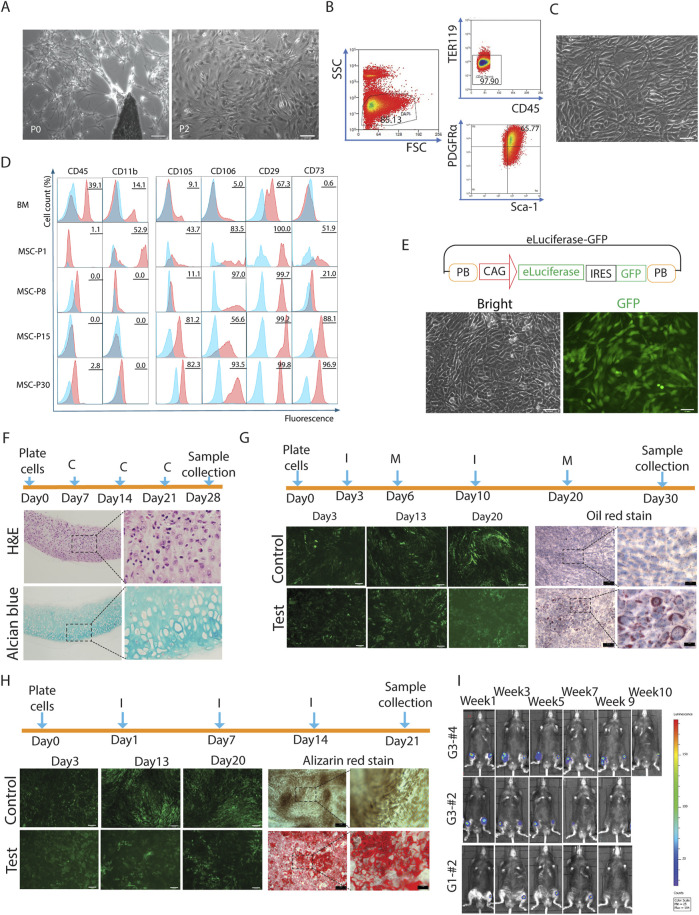
The generation of a mesenchymal stromal cell line. **(A)** Bone chips were seeded in the plate, and several days later, cells grew out from the bone chip (P0, magnification ×5), the cells were a heterogenous population (P2, magnification ×10); **(B)** Primary cultured cells were bunk sorted according to the expression of negative markers TER119 and CD45, and both PDGFRα and Scal-1 positive, the sorting gate was defined by isotype antibody controls; **(C)** The sorted cells looked more homogenous (magnification ×10); **(D)** The MSC negative and positive marker expressions on MSCs with different passages; **(E)** The components of e-Luciferase expression plasmid based on PB transposon system, which contained a GFP tag, the e-luciferase-GFP transfected MSC still kept the spindle morphology (magnification ×10); **(F)** The e-luciferase-GFP transfected MSCs were differentiated to chondrocytes (stained by Alcian blue) (magnification ×5), C-complete medium; **(G)** The e-luciferase-GFP transfected MSCs were differentiated to adipocytes (stained by Oil red) (magnification ×5), I-induction medium, M-maintenance medium; **(H)** The e-luciferase-GFP transfected MSCs were differentiated to osteocytes (stained by Alizarin red) (magnification ×5), I-induction medium; **(I)** 5 × 10^4^ of e-luciferase-GFP transfected MSCs were injected into C57BL6/J mouse joints, and monitored by luciferase-Luciferin reaction using BLI.

MSCs are a heterogeneous population of cells with self-renewal properties and the multipotential ability to differentiate into mesodermal cells, such as adipocytes, chondrocytes, and osteocytes (Pittenger et al., 1999). Injection of MSCs to the OA knee joint have surfaced as a promising cell-based therapy (Harrell et al., 2019). To establish a joint-friendly MSC cell line, we followed Zhu’s protocol to generate a cell line from mouse compact bones (Zhu et al., 2010). This resulted in a heterogeneous population of cells with various morphologies, such as spindle, stellate, and polygonal shapes (Figure 1A, P0, P2).

To make the cell population more homogeneous and enriched with proliferative and active cells, we further sorted the cells against negative MSC markers CD45 and TER119, then further sorted CD45^−^TER119^-^ cells by their expression of PDGFRα and Sca-1, finally, we collected the CD45^−^TER119^−^PDGFRα^+^Sca-1^+^ populations (Morikawa et al., 2009) (Figure 1B). After sorting, the cells appeared much more homogeneous (Figure 1C). To investigate whether MSC marker expression changed after multiple passages, we characterized MSC marker expression in passage 8, 15, and 30, and compared them with unsorted passage 1 cells, using bone marrow cells as a control (Figure 1D). Most of the MSC markers were stably expressed even up to passage 30.

To observe the morphological changes of the cells and track them after injection into mouse knees, we generated a *PiggyBac*-based e-Luciferase expression plasmid tagged with eGFP to transfect the MSCs. The engineered MSCs (transfected MSCs) were bulk sorted based on eGFP expression (Figure 1E). We then further tested the multilineage differentiation ability of these eLuciferase-eGFP engineered MSCs by differentiating them into chondrocytes, adipocytes, and osteocytes (Figures 1F–H). For chondrocyte differentiation, pellets were collected at day 28 following the protocol provided by the manufacturer and H&E and Alcian blue stains were used to confirm the presence of chondrocytes (Figure F). For adipocyte differentiation, engineered cells at 100% confluence were cultured with 3 cycles of induction/maintenance medium, while control cells were cultured in normal MSC culture medium. Cells were stained with Oil Red and numerous lipid droplets were observed in the differentiation group (Figure 1G). For osteocyte differentiation, induction medium was used for the entire differentiation protocol, while control cells were maintained in normal MSC culture medium. By day 21, Alizarin red staining was used and revealed the morphology of osteocytes (Figure 1H).

To explore how long MSCs can survive in the mouse knees, we injected 5 × 10^4^ MSCs into mouse knees and tracked the cells by intraperitoneal injection of luciferin, monitoring the local signal using BLI (Figure 1I). Interestingly, most of the injected cells survived in the knee joint for up to 7 weeks, with the longest survival reaching up to 72 days. Injected cells were not observed in other body parts of the mouse.

**FIGURE 2:**
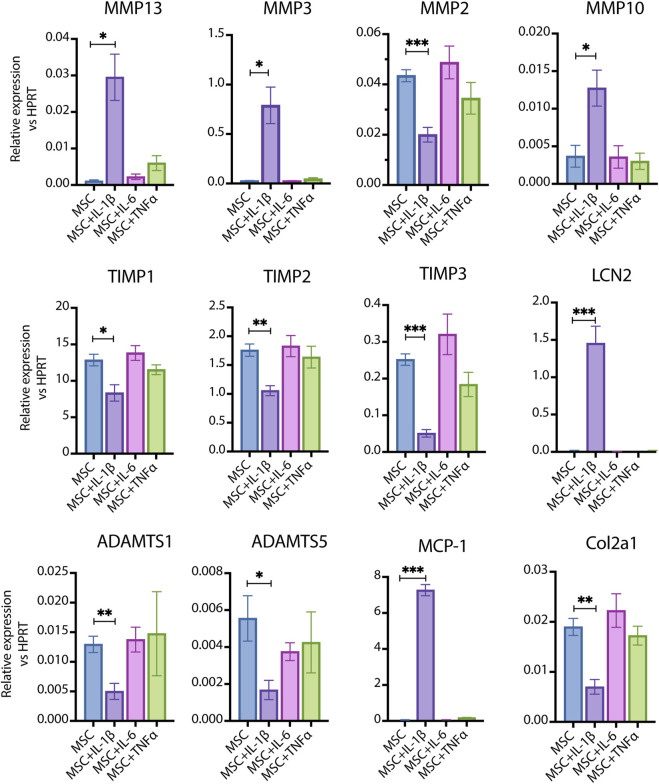
The response of primary cultured MSCs to pro-inflammatory cytokines. 100 pg/mL of IL-1β, IL-6 and TNF-α were used to stimulate MSCs respectively. IL-1β significantly induced the expression of MMP13, MMP3, MMP10, LCN2 and MCP-1 (IL-1β vs. Control, p < 0.05–0.001, n = 4–6); IL-1β significantly inhibited the expression of MMP2, TIMP1, TIMP2, TIMP3, ADAMTS1, ADAMTS5 and Col2a1(IL-1β vs. Control, p < 0.05–0.001, n = 4–6); while the same dosage of IL-6 and TNF-α did not induce significant changes. Data are expressed as mean ± SEM, *p < 0.05; **p < 0.01; ***p < 0.001.

MSCs, which serve as precursors to chondrocytes, play a pivotal role in the initiation and progression of OA. To investigate how compact bone-derived MSCs respond to pro-inflammatory cytokines, we stimulated them with IL-1β at a concentration of 100 pg/mL and compared their responses to those induced by interleukin-6 (IL-6) and tumor necrosis factor-alpha (TNF-α) at the same dosage (Figure 2). IL-1β significantly increased the expression of tissue matrix metalloproteinases (MMPs), including MMP13, MMP3, and MMP10, while notably reducing the expression of MMP inhibitors such as tissue inhibitor of metalloproteinase 1 (TIMP1), TIMP2, and TIMP3. Additionally, it suppressed MMP2, A disintegrin and metalloproteinase with thrombospondin motifs 1 (ADAMTS1), ADAMTS5, and collagen type II alpha 1 (COL2A1). Collectively, these changes promote catabolic processes associated with OA progression. Interestingly, IL-1β also significantly elevated the levels of Lipocalin 2 (LCN2) and monocyte chemoattractant protein 1 (MCP-1). The relevance of the LCN2 increase is further explored in our other paper (Biao Li et al., submitted for publication).

### 3.2 Generation of soluble IL1R2 trap (sIL1R2) and sticky IL1R2 trap (stkIL1R2)

**FIGURE 3:**
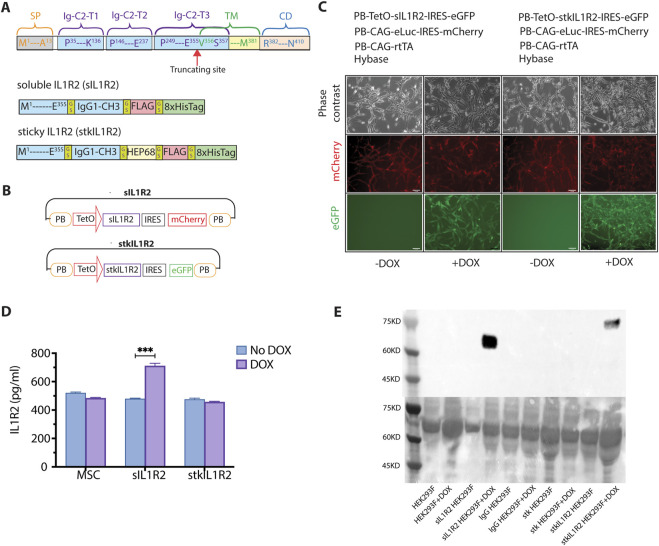
The generation of sticky IL-1 receptor 2 (stkILR2) biologics and stable expression cell lines. **(A)** The strategy and the primary components of ILR2, sIL1R2 and stkIL1R2; **(B)** The expression plasmids of sIL1R2 and stkIL1R2 based on PB transposon system, which included a TetO operator; **(C)** sIL1R2 and stkIL1R2 plasmids were transfected into MSCs, the expression of sIL1R2 and stkIL1R2 were under the control of Dox (magnification ×5); **(D)** In the presence of Dox, there was a significant increase of sIL1R2 in the supernatant quantified by ELISA (No Dox vs. Dox, p < 0.001, n = 3), while there was significant expression of stkIL1R2 in the supernatant; **(E)** Forced expression of sIL1R2 and stkIL1R2 in HEK293 cells detected by Western blot assay, the protein samples were from the supernatants in the presence of Dox. Data are expressed as mean ± SEM, ***p < 0.001.

IL1R2 is a transmembrane protein that consists of three key domains: an intracellular domain (spanning residues R382 to N410), a transmembrane domain (residues V356 to M381), and an extracellular domain (residues M1 to E355) (Figure 3A). The extracellular domain includes three Ig-C2 motifs responsible for ligand binding. To generate a sIL1R2 while maintaining optimal binding efficiency to its ligands, we truncated the extracellular domain at the E355-V356 boundary, using the soluble fragment from M1 to E355 to develop our biologic.

The final sIL1R2 biologic comprises the IgG1-CH3 domain, a FLAG tag, and eight His tags, all connected by GS linkers (Figure 3A). In comparison, the stkIL1R2 contains an additional cassette, including a heparin-binding domain (HBD) encoded by VEGF-A exon 6 and a non-HBD encoded by VEGF-A exon 8 (Michael et al., 2014). This additional domain is flanked by GS linkers and inserted between the IgG1-CH3 domain and the FLAG tag of sIL1R2 (Figure 3A).

A Dox-inducible gene expression cassette, utilizing the Tet-ON system, was employed to enable inducible expression (Das et al., 2016). The plasmids for inducible expression were constructed using the *PiggyBac* (PB) transposon system. In this system, the upstream promoter cassette, TetO, is activated following the binding of rtTA in the presence of Dox, it triggers the downstream fluorescence signal, with mCherry used for sIL1R2 and GFP for stkIL1R2 (Figure 3B).

To create MSC lines for injection, an inducible stkIL1R2 engineered MSC line was generated by co-transfecting PB-TetO-stkIL1R2-IRES-eGFP and PB-CAG-eLuc-IRES-mCherry plasmids into MSCs. An sIL1R2 MSC line was also generated as a control (Figure 3C). For cells stably expressing sIL1R2 or stkIL1R2, supernatants were collected and analyzed using an ELISA kit designed to detect the extracellular domain of IL1R2. With Dox induction, ELISA detected the expression of sIL1R2 in the engineered MSC supernatant and did not detect the expression of stkIL1R2 in the supernatant (Figure 3D).

As we did not detect the expression of stkIL1R2 in the transgenic MSC supernatant (Figure 3D), and we wanted to confirm the presence of stkIL1R2 protein, we resorted to the protein generation platform HEK293 cells. sIL1R2 and stkIL1R2 expression plasmids were transfected into the HEK293F protein production cell line to force the production of proteins. IgG and stk plasmids were used and served as controls. The collected supernatants were analyzed by Western blot to detect the protein forms (Figure 3E). We can see there were clear protein expression of sIL1R2 and stkIL1r2 proteins, which means our transgenes did generate proteins.

### 3.3 *In vitro* functional test of stkIL1R2

**FIGURE 4:**
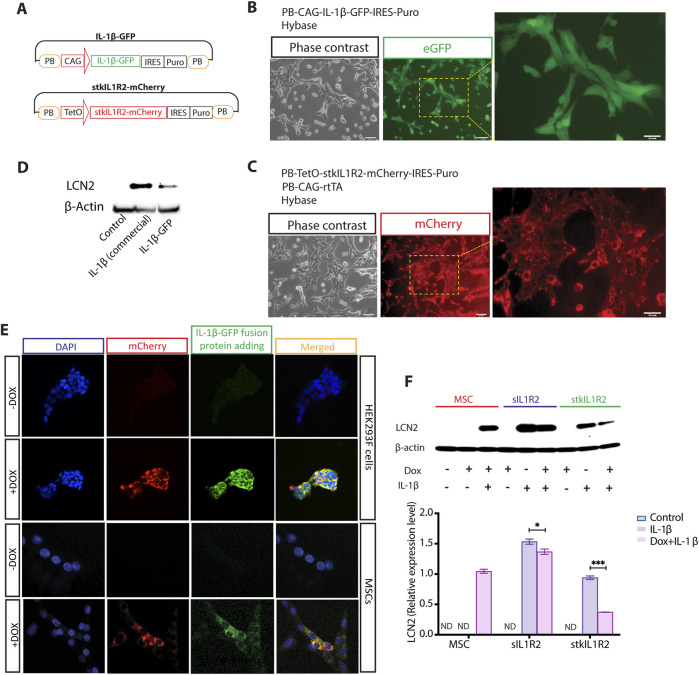
*In vitro* functional test of stkIL1R2. **(A)** The components of IL-1β-GFP fusion protein gene and stkIL1R2-mCherry fusion protein gene expression plasmids based on PB transposon system; **(B)** The expression of IL-1β-GFP fusion protein in MSCs, which showed an evenly distribution in the cytoplasm (magnification ×10); **(C)** The expression stkIL1R2-mCherry fusion protein in MSCs looked like clumps in the cells, which was obviously different from the expression pattern of IL-1β-GFP fusion protein (magnification ×10); **(D)** The home-made IL-1β-GFP fusion protein had similar function the commercial IL-1β in inducing LCN2 expression, which is a specific biomarker of IL-1β; **(E)** stkIL1R2-mCherry fusion gene transfected HEK293F cells and MSCs were plated on slides with and without Dox respectively, cells were fixed without perforated regents, then IL-1β-GFP fusion protein were added to each, then washed out with PBS, the binding of IL-1β-GFP fusion protein on the cell membrane and co-localized with mCherry signal clearly demonstrated that stkIL1R2 was on the cell membrane and ECM (magnification ×5); **(F)** Western blotting of IL-1β biomarker LCN2 expression in MSCs showed that both sIL1R2 and stkIL1R2 significantly blocked the function of 100 pg/mL of IL-1β (For sIL1R2, Dox + vs. Dox-, p = 0.0198, n = 3; For stkIL1R2, Dox + vs. Dox-, p = 0.0004, n = 3). Data are expressed as mean ± SEM, *p < 0.05; ***p < 0.001.

To visualize the binding ability of stkIL1R2 with IL-1β and demonstrate that stkIL1R2 protein adheres to the cell surface and ECM, we developed a novel binding test platform. First, we generated a fusion protein IL-1β-GFP gene expression plasmid (PB-CAG-IL-1β-GFP-IRES-puro-PB) and an inducible fusion protein stkIL1R2-mCherry gene expression plasmid (PB-TetO-stkIL1R2-mCherry-IRES-puro-PB) (Figure 4A).

Both plasmids were transfected into MSCs, and after puromycin selection, most MSCs expressed the target proteins. Interestingly, the IL-1β-GFP protein exhibited an even distribution of GFP signal within the cells (Figure 4B), whereas the stkIL1R2-mCherry protein displayed an entirely different distribution pattern. It appeared as variably sized dots randomly scattered throughout the cell surface and ECM (Figure 4C).

To produce IL-1β-GFP fusion protein, the IL-1β-GFP plasmid was transfected into HEK293F cells, and the IL-1β-GFP fusion protein was isolated from the supernatant. Functionally, the IL-1β-GFP fusion protein was comparable to commercial IL-1β in inducing LCN2 expression in MSCs (Figure 4D). Subsequently, the stkIL1R2-mCherry plasmid was transfected into both HEK293F cells and MSCs (Figure 4E). Following Dox induction, stkIL1R2-mCherry expression appeared as random nuclear and cytoplasmic dots in the cells. The cells were fixed without permeabilization reagents, and purified IL-1β-GFP fusion protein was added to the surface of fixed cells. In the absence of Dox induction, there was little to none binding of IL-1β-GFP fusion protein to the cells. However, when Dox was present, there was significant binding of IL-1β-GFP fusion protein to the cells, with GFP signals co-localizing strongly with mCherry signals. This strategy clearly demonstrated that stkIL1R2 adhered to the cell membrane and extracellular matrix (ECM) and effectively bound to IL-1β.

To further confirm the binding ability and function of stkIL1R2, we evaluated the response of stkIL1R2-transfected MSCs to IL-1β stimulation. Unstimulated MSCs and Dox added MSC were served as negative controls, while MSCs stimulated with 100 pg/mL of IL-1β served as positive controls. For both sIL1R2 and stkIL1R2, IL-1β induced a notable expression of LCN2 compared to positive control, while under the induction of Dox, sIL1R2 expression significantly reduced the expression of LCN2 induced by IL-1β (No Dox vs. Dox, p < 0.05, n = 3); Dox induced expression of stkIL1R2 also significantly reduced the expression of LCN2 induced by IL-1b (No Dox vs. Dox, p < 0.001, n = 3) (Figure 4F).

### 3.4 *In vivo* evaluation of stkIL1R2

**FIGURE 5:**
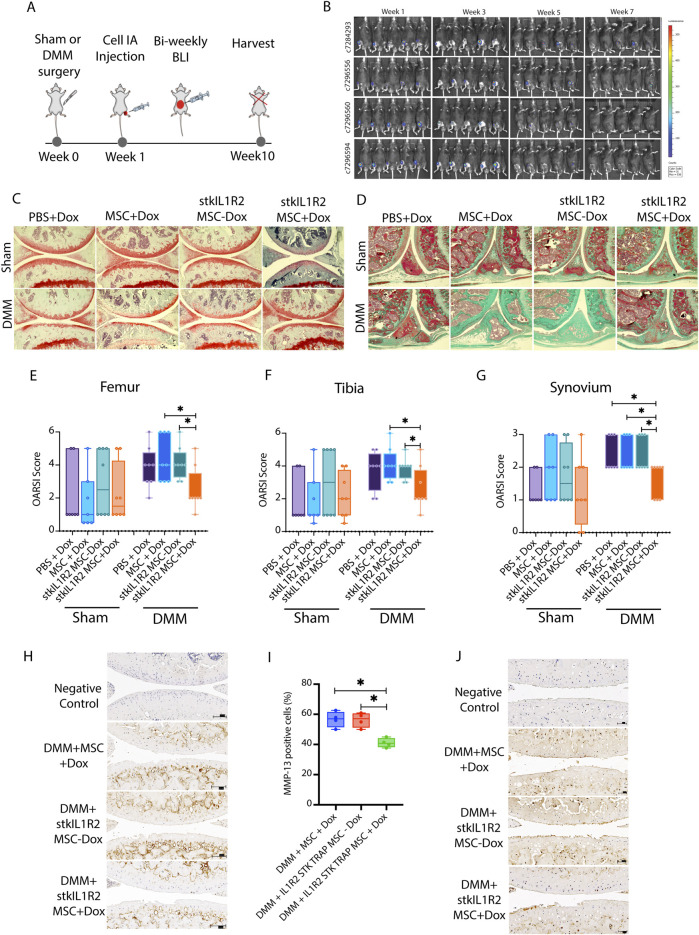
MSC expressed stkIL1R2 attenuated the progression of OA in DMM. **(A)** The study design of *in vivo* test of stkIL-1R2; **(B)** The BLI monitoring results from stkIL-1R2 expression MSCs injected into the joint cavities; **(C)** The histological results of femur and tibia from Sham and DMM groups after 10 weeks (magnification ×10); **(D)** The histological results of synovium from Sham and DMM groups after 10 weeks (magnification ×10); **(E)** Quantification of the pathological changes by OARSI score on femur, stkIL1R2 expression significantly reduced the OARSI scores in femur from DMM group (stkIL1R2 MSC + Dox vs. MSC + Dox and stkIL1R2 MSC-Dox, p < 0.05, n = 8 for each); **(F)** Quantification of the pathological changes by OARSI score on tibia, stkIL1R2 expression significantly reduced the OARSI scores in tibia from DMM group (stkIL1R2 MSC + Dox vs. MSC + Dox and stkIL1R2 MSC-Dox, p < 0.05, n = 8 for each); **(G)** Quantification of the pathological changes by OARSI score on synovium, stkIL1R2 expression significantly reduced the OARSI score in synovium (stkIL1R2 MSC + Dox vs. PBS + Dox, MSC + Dox and stkIL1R2 MSC-Dox respectively, p < 0.05, n = 8 for each); **(H)** IHC staining of the expression of MMP-13 on femur and tibia (magnification ×10); **(I)** Quantification of MMP-13 expression cells on both femur and tibia of DMM groups, stkIL1R2 significantly reduced the expression of MMP-13 (stkIL1R2 MSC + Dox vs. MSC + Dox and stkIL1R2 MSC-Dox, p < 0.05, n = 4 for each); **(J)** IHC staining of C1,C2 expression on femur and tibia of DMM groups (magnification ×10). Data are expressed as mean ± SDM, *p < 0.05. IA: Intraarticular.

To examine the efficiency of the stkIL1R2 trap in slowing the progression of OA, we utilized the destabilization of the murine DMM model, a well-established post-traumatic OA (PTOA) model. The study groups were as follows: 1) Sham + PBS + Dox; 2) Sham + MSC + Dox; 3) Sham + stkIL1R2 MSC–Dox; 4) Sham + stkIL1R2 MSC + Dox; 5) DMM + PBS + Dox; 6) DMM + MSC + Dox; 7) DMM + stkIL1R2 MSC–Dox; 8) DMM + stkIL1R2 MSC + Dox. One week after surgery, phosphate-buffered saline (PBS), 5 × 10^4^ MSCs, or stkIL1R2 expressing MSCs were injected intra-articular into the right knees of mice. Dox chow was administered to the Dox + groups. At 10 weeks post-surgery, all mice were sacrificed, and their knees were collected for analysis (Figure 5A).

BLI of luminescent signals from luciferin-luciferase reaction in groups 3, 4, 7, and 8 was monitored for up to 7 weeks (Figure 5B). Representative images of safranin-O-stained sections from sham-only and DMM-only groups were analyzed (Supplementary Figure S1). Compared to sham-only sections (Supplementary Figures 1A1–5), DMM-only sections (Supplementary Figures 1B1–5) exhibited loss of safranin-O staining, cartilage erosion, vertical clefts, and lesions extending to the calcified layer, affecting 25%–50% of the articular cartilage. Moderate to severe synovitis was also observed (Supplementary Figures 1B1–4). OARSI scoring for the femur, tibia, and synovium confirmed that the DMM-only group had significantly higher scores for OA damage and synovitis compared to the sham-only group (Supplementary Figures 1C–E), validating the OA changes induced in the DMM group.

Next, we assessed the efficacy of the stkIL1R2 trap in slowing OA progression in the DMM model. No significant differences were observed in the severity of cartilage degeneration or synovitis among the sham groups (Figures 5C,D, Sham), which was confirmed by the quantification using OARSI score (Figures 5E–G, Sham). On the other side, in both of the femur and tibia of DMM groups (Figures 5E,F, 5DMM), DMM + stkIL1R2 MSC + Dox significantly attenuated DMM-induced cartilage degeneration as quantified by OARSI scoring, compared to the DMM + stkIL1R2 MSC–Dox and DMM + MSC + Dox groups. Synovitis was also significantly reduced in the DMM + stkIL1R2 MSC + Dox group compared to all other DMM groups (Figures 5G,DMM).

To confirm the effectiveness of our new biologics in OA treatment, we evaluated cartilage breakdown markers: MMP-13 and collagen type I/II cleavage neoepitope (C1,2C). Immunohistochemical stained sections for MMP-13 (Figures 5H,I) and C1,2C (Figure 5J) showed a significant decrease in the percentage of MMP-13-positive cells and C1,2C expression in the DMM + stkIL1R2 MSC + Dox group compared to the DMM + stkIL1R2 MSC–Dox and DMM + MSC + Dox groups.

## 4 Discussion

In this study, we developed an inducible, locally acting IL-1β sticky-trap (stkIL1R2) plasmid based on the PiggyBac transposon system and transfected it into murine MSCs derived from compact bone. This approach established a cell-based gene expression therapeutic platform, which was tested for its efficacy using the murine DMM model. Our findings demonstrated that the IL-1β sticky-trap effectively delayed OA progression and reduced synovitis in the DMM model.

OA is increasingly recognized as a low-grade inflammatory whole-joint disease, with innate immunity playing a central role in its inflammatory pathology (Knights et al., 2023; Robinson et al., 2016; Roover et al., 2023; Scanzello, 2017b). Among pro-inflammatory cytokines, IL-1β has been well-documented to contribute to OA pathogenesis (Krenytska et al., 2023; Panina et al., 2017; Wang et al., 2019). However, anti-IL-1 strategies have produced inconsistent results. Given that IL-1β is a small molecule with activity in the picogram range (Gentile et al., 2013) and a short serum half-life, these challenges are likely due to spatial and temporal mismatches between IL-1β activity and the administered therapeutic agents (Kudo et al., 1990; REIMERS et al., 1991). To overcome this limitation, we employed a cell-based, locally expressed sticky biologics technology, generating MSCs that inducibly express stkIL1R2 biologics.

IL-1R2 is a decoy receptor with high binding affinity for both interleukin-1alpha (IL-1α) and IL-1β, surpassing that of IL-1 receptor type 1 (IL-1R1) (Mantovani et al., 1998). We harnessed this strong binding capacity, integrating IL-1R2 with the sticky-trap technology previously developed in our lab (Michael et al., 2014) to create the novel biologic stkIL1R2. This construct was introduced into compact bone-derived MSCs via an inducible expression plasmid. Fluorescent protein tagging confirmed that stkIL1R2 was expressed on the cell membrane and could bind IL-1β. Functional assays further demonstrated that MSCs expressing stkIL1R2 effectively blocked 50 pg/mL of IL-1β induced LCN2 expression.

To assess the therapeutic efficacy of stkIL1R2, we utilized the classical surgical OA model through the DMM, which mimics PTOA. In this model, transection of the medial meniscotibial ligament destabilizes the medial meniscus, leading to cartilage damage and subsequent OA (Glasson et al., 2007). At 10 weeks post-surgery, histological analysis of DMM joints revealed three key pathological changes: loss of cartilage integrity between the femur and tibia, thinning of subchondral bone, and significant inflammatory cell infiltration at the joint periphery. These findings were quantified using OARSI scores for the femur, tibia, and synovium, confirming the successful induction of moderate to severe OA, consistent with previous studies (Glasson et al., 2010).

In this study, we administered 5 × 10^4^ MSCs per injection into the murine knee joint. The survival of injected cells was monitored via BLI, revealing cell viability for up to 7 weeks. Histological analyses at 10 weeks post-surgery showed that the IL-1β sticky trap significantly reduced joint OA and synovitis compared to sham and other DMM control groups. These findings were corroborated by decreased expression of cartilage breakdown markers MMP-13 and collagen cleavage neoepitope C1,2C in the stkIL1R2 treated groups.

Despite these promising results, the study has some limitations. First, the compact bone-derived MSCs used were sorted in bulk, resulting in a heterogeneous cell population with variable reproducibility and biologic productivity. Second, the luciferase-luciferin tracking system has its limitations for monitoring injected cells in the knee joints as luciferin delivery to the cells may be low due to post traumatic changes to the knee joint and limited blood supply to the joint cavity, cartilage and parts of the meniscus. Also, the strength of the luminescence may need to be a stronger luciferase variant to detect and improve cell tracking in the knee joint. Lastly, the efficacy of stkIL1R2 was evaluated using a PTOA model, which may not fully represent other forms of OA. Future studies should explore its performance in chemically induced OA models or other relevant systems.

In conclusion, this study developed a novel, locally acting IL-1β sticky-trap (stkIL1R2) expressed in compact bone-derived MSCs. We demonstrated its binding ability to IL-1β and its therapeutic efficacy in preventing OA progression using the murine DMM model. The stkIL1R2 biologic shows strong potential as a stand-alone treatment or in combination with other therapeutic strategies for OA. These findings provide a foundation for new approaches to combat OA.

## Data Availability

The raw data supporting the conclusions of this article will be made available by the authors, without undue reservation.

## Supplementary material

SUPPLEMENTARY FIGURE S1
Generation of murine DMM OA model. **(A)** Sham mouse joint histology, 1–4 are 4 corners of the joint to show synovial membranes, 5 is the Zoom in figure of femur and tibia; **(B)** DMM mouse joint histology, 1–4 are 4 corners of the joint to show synovial membranes, 5 is the Zoom in figure of femur and tibia; **(C)** OARSI score was used to evaluate the histological changes after 10 weeks of surgery, DMM group OARSI score of femur was significantly higher than Sham groups (Sham vs. DMM, p < 0.05, n = 6,5); **(D)** DMM group OARSI score of tibia was significantly higher than Sham groups (Sham vs. DMM, p < 0.05, n = 6,5); **(E)** DMM group OARSI score of synovial membrane was significantly higher than Sham groups (Sham vs. DMM, p < 0.05, n = 6,5). Data are expressed as mean ± SEM, *p < 0.05; **p < 0.01; ***p < 0.001.
