# Supplementary material for: Engineered mesenchymal stromal cells with interleukin-1beta sticky-trap attenuate osteoarthritis in knee joints
Source: Front Cell Dev Biol. 2025 Apr 8;13:1559155. doi: 10.3389/fcell.2025.1559155 (PMC12011853; doi:10.3389/fcell.2025.1559155)
Supplement: Supplementary file 1 [file Table1.docx]

Table 1. Antibodies used in MSC sorting and characterizing

| Antibody name | Catalog number | Company |
| --- | --- | --- |
| CD45 | 103105 | Biolegend |
| TER119 | 116203 | Biolegend |
| PDGFRa | 323511 | Biolegend |
| Scal-1 | 108143 | Biolegend |
| CD11b | 101241 | Biolegend |
| CD105 | 120414 | Biolegend |
| CD106 | 105719 | Biolegend |
| CD29 | 102226 | Biolegend |
| CD73 | 127229 | Biolegend |
| DAPI | 422801 | Biolegend |
| Rat IgG 2b K | 400607 | Biolegend |
| Rat IgG 2a K | 400505 | Biolegend |
