# Supplementary figures and images for: Engineered mesenchymal stromal cells with interleukin-1beta sticky-trap attenuate osteoarthritis in knee joints
Source: Front Cell Dev Biol. 2025 Apr 8;13:1559155. doi: 10.3389/fcell.2025.1559155 (PMC12011853; doi:10.3389/fcell.2025.1559155)

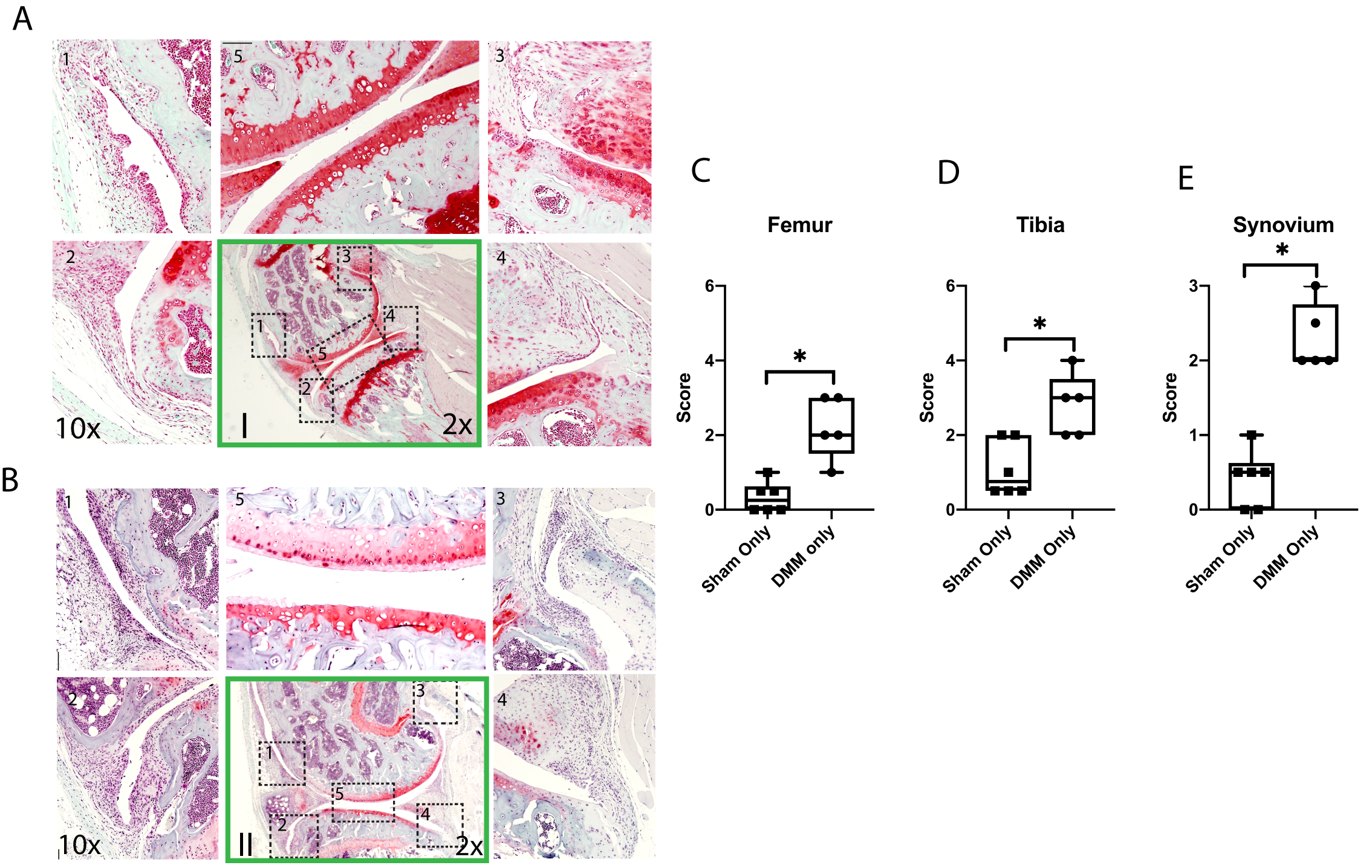

Supplement: Supplementary file 2 [file Image1.tif]
