# Supplementary material for: Engineered mesenchymal stromal cells with interleukin-1beta sticky-trap attenuate osteoarthritis in knee joints
Source: Front Cell Dev Biol. 2025 Apr 8;13:1559155. doi: 10.3389/fcell.2025.1559155 (PMC12011853; doi:10.3389/fcell.2025.1559155)
Supplement: Supplementary file 3 [file Table2.docx]

Table 2 qPCR primers used in this project

| Primer name | Forward | Reverse |
| --- | --- | --- |
| MMP13 | CTTCTTCTTGTTGAGCTGGACTC | CTGTGGAGGTCACTGTAGACT |
| MMP3 | ACATGGAGACTTTGTCCCTTTTG | TTGGCTGAGTGGTAGAGTCCC |
| MMP2 | ACCTGAACACTTTCTATGGCTG | CTTCCGCATGGTCTCGATG |
| MMP10 | GAGCCACTAGCCATCCTGG | CTGAGCAAGATCCATGCTTGG |
| TIMP1 | GCAACTCGGACCTGGTCATAA | CGGCCCGTGATGAGAAACT |
| TIMP2 | TCAGAGCCAAAGCAGTGAGC | GCCGTGTAGATAAACTCGATGTC |
| TIMP3 | CTTCTGCAACTCCGACATCGT | GGGGCATCTTACTGAAGCCTC |
| ADAMTS1 | CATAACAATGCTGCTATGTGCG | TGTCCGGCTGCAACTTCAG |
| ADAMTS5 | GGAGCGAGGCCATTTACAAC | CGTAGACAAGGTAGCCCACTTT |
| MCP1 | TTAAAAACCTGGATCGGAACCAA | GCATTAGCTTCAGATTTACGGGT |
| Col2a1 | GGGAATGTCCTCTGCGATGAC | GAAGGGGATCTCGGGGTTG |
| LCN2 | TGGCCCTGAGTGTCATGTG | CTCTTGTAGCTCATAGATGGTGC |
| mHprt | GGCTATAAGTTCTTTGCTGAC | AACTTTTATGTCCCCCGTTGA |
